# Association of labour duration in spontaneous deliveries with low neonatal Apgar scores and foetal acidosis: the Japan Environment and Children’s Study

**DOI:** 10.1038/s41598-022-24359-3

**Published:** 2022-12-13

**Authors:** Tsuyoshi Murata, Shun Yasuda, Karin Imaizumi, Hirotaka Isogami, Toma Fukuda, Hyo Kyozuka, Akiko Yamaguchi, Akiko Sato, Yuka Ogata, Kosei Shinoki, Mitsuaki Hosoya, Seiji Yasumura, Koichi Hashimoto, Hidekazu Nishigori, Keiya Fujimori, The Japan Environment, Children’s Study (JECS) Group, Michihiro Kamijima, Shin Yamazaki, Yukihiro Ohya, Reiko Kishi, Nobuo Yaegashi, Koichi Hashimoto, Chisato Mori, Shuichi Ito, Zentaro Yamagata, Hidekuni Inadera, Takeo Nakayama, Hiroyasu Iso, Masayuki Shima, Hiroshige Nakamura, Narufumi Suganuma, Koichi Kusuhara, Takahiko Katoh

**Affiliations:** 1 Fukushima Regional Center for the Japan Environment and Children’s Study, Fukushima, Japan; 2 Department of Obstetrics and Gynecology, Fukushima Medical University School of Medicine, 1 Hikarigaoka, Fukushima, 960-1295 Japan; 3 Department of Pediatrics, Fukushima Medical University School of Medicine, Fukushima, Japan; 4 Department of Public Health, Fukushima Medical University School of Medicine, Fukushima, Japan; 5 Fukushima Medical Center for Children and Women, Fukushima Medical University, Fukushima, Japan; 6 Nagoya City University, Nagoya, Japan; 7 National Institute for Environmental Studies, Tsukuba, Japan; 8 National Center for Child Health and Development, Tokyo, Japan; 9 Hokkaido University, Sapporo, Japan; 10 Tohoku University, Sendai, Japan; 11 Fukushima Medical University, Fukushima, Japan; 12 Chiba University, Chiba, Japan; 13 Yokohama City University, Yokohama, Japan; 14 University of Yamanashi, Chuo, Japan; 15 University of Toyama, Toyama, Japan; 16 Kyoto University, Kyoto, Japan; 17 Osaka University, Suita, Japan; 18 Hyogo College of Medicine, Nishinomiya, Japan; 19 Tottori University, Yonago, Japan; 20 Kochi University, Nankoku, Japan; 21 University of Occupational and Environmental Health, Kitakyushu, Japan; 22 Kumamoto University, Kumamoto, Japan

**Keywords:** Medical research, Epidemiology

## Abstract

This study evaluated the association between labour duration (LD) and incidence of low neonatal Apgar scores and foetal acidosis. Data of 37,682 women with full-term singleton spontaneous vaginal deliveries from the Japan Environment and Children’s Study were analysed. Women were classified according to the median LD as nulliparous (< 10 or ≥ 10 h) or multiparous (< 5 or ≥ 5 h) and further into five subcategories: nulliparous (< 10.0, 10.0–12.9, 13.0–15.9, 16.0–18.9, and ≥ 19 h) and multiparous (< 5.0, 5.0–7.9, 8.0–10.9, 11.0–13.9, and ≥ 14.0 h). Multiple logistic regression models were used to determine odds ratios (ORs) for outcomes in women with over-median LD. Over-median LD exhibited no statistically significant association with low neonatal Apgar scores. The adjusted ORs for both umbilical artery (UmA-pH) < 7.2 and < 7.1 were increased in nulliparous women with over-median LD, whereas only the adjusted OR for UmA-pH < 7.2 was increased in multiparous women with over-median LD. Moreover, this association manifested as a plateau in nulliparous women with LD ≥ 13 h and without dose-dependent association in multiparous women.

## Introduction

The Apgar score provides an accepted and convenient method for reporting the status of new-born infants immediately after birth and the response to resuscitation if needed^1^. Although the Apgar score should not be used for predicting individual neonatal mortality or neurologic outcomes, it is partially associated with neonatal mortality and neurological outcomes^1^; low neonatal Apgar scores are generally defined as < 7^1^. Moreover, foetal and neonatal asphyxia significantly contribute to neonatal morbidity and mortality^2^. Specifically, foetal acidosis is associated with birth asphyxia resulting from the interruption of placental blood flow and subsequent foetal hypoxia and hypercarbia^2–4^. Low umbilical artery pH (UmA-pH) is significantly associated with neonatal mortality^3^, whereas metabolic acidosis is associated with foetal hypoxic-ischaemic brain injury^5^. Thus, intrapartum foetal assessments have been refined to increase the detection of foetal acidosis and to decrease its incidence^6^.

Low neonatal Apgar scores and foetal acidosis are reportedly associated with several perinatal factors^7,8^, and the labour environment around the foetus is closely associated with the incidence of low neonatal Apgar scores and foetal acidosis. Prolonged labour duration (LD) affects the incidence of neonatal morbidity^9,10^ and results in low Apgar scores^10^. However, several studies have indicated that a prolonged first stage of labour is not associated with an increased incidence of adverse neonatal outcomes^11,12^ and that a prolonged second stage of labour is not associated with an increased incidence of low neonatal Apgar scores and foetal acidosis^13–15^. Detailed analysis regarding the association between prolonged LD and the incidence of low neonatal Apgar scores and foetal acidosis in stratified analyses based on parity, usage of operative deliveries, and presence of labour induction and augmentation using a nationwide population is lacking. Moreover, the proper LD in different labour situations remains unclear.

This study aimed to analyse the association between LD and the incidence of low neonatal Apgar scores and foetal acidosis in Japanese women with spontaneous uncomplicated deliveries, who have a leaner physique than women in western countries^7,8^, and to determine the cut-off LD value to identify the risk of foetal acidosis and reduce the incidence of foetal adverse outcomes. We comprehensively analysed both low neonatal Apgar scores and foetal acidosis since these have different aetiologies as well as related conditions and outcomes^1–6^. We hypothesised that longer LD is associated with increased incidence of low neonatal Apgar scores and foetal acidosis even in Japanese women, given that prolonged LD affects the incidence of neonatal morbidity^9,10^. Thus, we focused on the association of over-median LD with the incidence of low neonatal Apgar scores and foetal acidosis in nulliparous and multiparous women.

## Methods

### Study design

We analysed the data from the Japan Environment and Children’s Study (JECS), a nationwide, government-funded, prospective birth cohort study that started in January 2011 (participants enrolled during January 2011–March 2014) to investigate the effects of environmental factors on children’s health^16,17^. Details about the JECS have been provided elsewhere^16,17^. Some studies have reported associations between pregnancy factors and offspring outcomes^7,8^. Moreover, as the JECS is a prospective birth cohort study, future studies will clarify variable associations between prenatal and postnatal factors and children’s health.

Participants were recruited at the first prenatal examination at cooperating health care providers or at local government offices that issued a pregnancy journal, the Maternal and Child Health Handbook, to all expectant mothers in Japan before they received municipal services for pregnancy, delivery, and childcare. Pregnant women were contacted through the cooperating health care providers and/or local government offices issuing Maternal and Child Health Handbooks, and those willing to participate were registered^16,17^. Self-administered questionnaires, completed by the women during the first and second/third trimesters, were used to collect information on demographic factors, medical history, physical and mental health, lifestyle, occupation, environmental exposures at home and in the workplace, housing conditions, and socioeconomic status^16,17^.

### Data collection

The current analysis used data released in October 2019 (dataset: jecs-ta-20190930). Women with singleton pregnancies were included, and women with abortions; stillbirths; preterm births before 37 weeks of gestation; caesarean deliveries; operative vaginal deliveries; foetal presentation anomalies; epidural analgesia use during labour; missing information regarding exposure, outcomes, and confounding factors; and usage of labour induction and augmentation were excluded to analyse the association between at-term spontaneous vaginal deliveries without labour induction and augmentation and the incidence of low neonatal Apgar scores and foetal acidosis. The status of these variables was derived from medical record transcripts.

### Exposure variables

LD was defined as the duration from labour onset (regular uterine contractions with pain every 10 min or six regular uterine contractions with pain in a 1-h period) to delivery. LD was derived from medical record transcripts. Women were stratified based on parity (nulliparous and multiparous) and median LD (nulliparous women, 573.00 min; multiparous women, 288.00 min). In stratification (1), women were divided based on parity and further divided according to two LD categories: nulliparous women (< 10 h or ≥ 10 h) and multiparous women (< 5 h or ≥ 5 h). In stratification (2), women were divided based on parity and further divided according to five LD categories (dividing over-median LD into four categories): nulliparous women (< 10.0, 10.0–12.9, 13.0–15.9, 16.0–18.9, and ≥ 19 h) and multiparous women (< 5.0, 5.0–7.9, 8.0–10.9, 11.0–13.9, and ≥ 14.0 h). We consistently set the under-median LD as the reference to analyse the association of over-median LD with the incidence of outcomes.

### Main outcome measures and confounding factors

The main outcome measure was the incidence of low neonatal Apgar scores and foetal acidosis, determined from medical record transcripts. Low neonatal Apgar scores are defined as < 7^1^. Because Apgar scores at 1 and 5 min have different dimensions of reflecting neonatal condition^1^, both of them were set as the main outcomes. Foetal acidosis was defined as UmA-pH < 7.2 or < 7.1^7,8^. These thresholds were chosen based on a study reporting that UmA-pH < 7.2 increased the risk of short-term neonatal adverse outcomes^18^ and another study reporting that UmA-pH < 7.1 increased the risk of neonatal adverse neurological sequelae^4^. We did not analyse the data regarding women with Apgar scores ≤ 3 because the number of women in this group was considerably small (0.2% in nulliparous women and 0.1% in multiparous women, both for 1- and 5-min Apgar scores, respectively). Similarly, we did not analyse data regarding women with UmA-pH < 7.0 because the number of women in this group was considerably small (0.2% in nulliparous women and 0.1% in multiparous women).

Maternal age, pre-pregnancy body mass index (BMI), gestational weight gain (GWG), maternal smoking status, maternal alcohol consumption status, maternal educational status, annual household income, neonatal birthweight, and presence of hypertensive disorders of pregnancy were considered potential confounding factors^7,8^. There was no multicollinearity, which was judged to present under the following conditions: an association between independent variables with a correlation coefficient of r > 0.8 and/or a variance inflation factor > 10.

GWG was calculated as the bodyweight just before delivery (retrieved from medical record transcripts) minus the bodyweight before pregnancy (kg)^7^. Participants were requested to provide information regarding their smoking status by choosing one of the following: ‘currently smoking’, ‘never’, ‘previously did, but quit before realising current pregnancy’, and ‘previously did, but quit after realising current pregnancy’^7,8^. Participants who responded ‘currently smoking’ were included in the ‘smoking’ category and others were included in the ‘non-smoking’ categories. Women were requested to provide information about their alcohol consumption status by choosing one of the following options: ‘never drank’, ‘quit drinking before pregnancy’, ‘quit drinking during early pregnancy’, and ‘kept drinking during pregnancy’^19^. Women who chose ‘kept drinking during pregnancy’ were grouped in the drinking category, and all other women were grouped in the non-drinking category. Maternal educational status was categorised into four groups based on the completed years of education (junior high school, < 10 years; high school, 10–12 years; technical junior college, technical/vocational college, associate degree, or bachelor’s degree, 13–16 years; and graduate degree, ≥ 17 years)^7,8^. Annual household income was categorised into four levels (< 2,000,000, 2,000,000–5,999,999, 6,000,000–9,999,999, and ≥ 10,000,000 JPY)^7,8^. Data on neonatal birthweight were derived from medical record transcripts. Hypertensive disorders of pregnancy were defined as persistently elevated blood pressure (≥ 140/90 mmHg) after 20 weeks of pregnancy in an otherwise normotensive woman^20^.

### Statistical analysis

Women were stratified based on parity and LD, and maternal characteristics and obstetric outcomes were compared.

Multiple logistic regression models were used to calculate the crude odds ratios (cORs), adjusted ORs (aORs), and 95% confidence intervals (CIs) for low neonatal Apgar scores (Apgar scores < 7 at 1 min or 5 min after birth) and foetal acidosis (UmA-pH < 7.2 or < 7.1) in women in each group in stratification (1). Multiple logistic regression models were used to calculate the cORs, aORs, and 95% CIs for foetal acidosis in women in each group in stratification (2). Women with under-median LDs were consistently used as a reference group. ORs were adjusted for the confounding factors mentioned earlier.

Moreover, multiple logistic regression models were used to calculate the ORs and 95% CIs for foetal acidosis in women in divided categories of under-median LD: nulliparous women (< 2.5, 2.5–4.9, 5.0–7.4, 7.5–9.9, and ≥ 10 h) and multiparous women (< 1.25, 1.25–2.4, 2.5–3.74, 3.75–4.9, and ≥ 5.0 h). Women with over-median LDs were used as a reference group in these comparisons.

Additionally, receiver operating characteristic curve analysis was performed to calculate the cut-off LD values for the prediction of the incidence of UmA-pH < 7.2 in nulliparous and multiparous women.

Statistical analysis was performed using SPSS version 26 (IBM Corp., Armonk, NY, USA), with *P* < 0.05 being considered statistically significant.

### Ethics declarations

The JECS protocol was reviewed and approved by the Ministry of the Environment Institutional Review Board on Epidemiological Studies (No. 100910001) and the Ethics Committees of all participating institutions. The JECS was conducted in accordance with the Helsinki Declaration and other national regulations and guidelines. Written informed consent was obtained from all participants. Informed consent was obtained from a parent or a legal guardian for participants below 20 years old.

## Results

### Study participants

**Figure 1:**
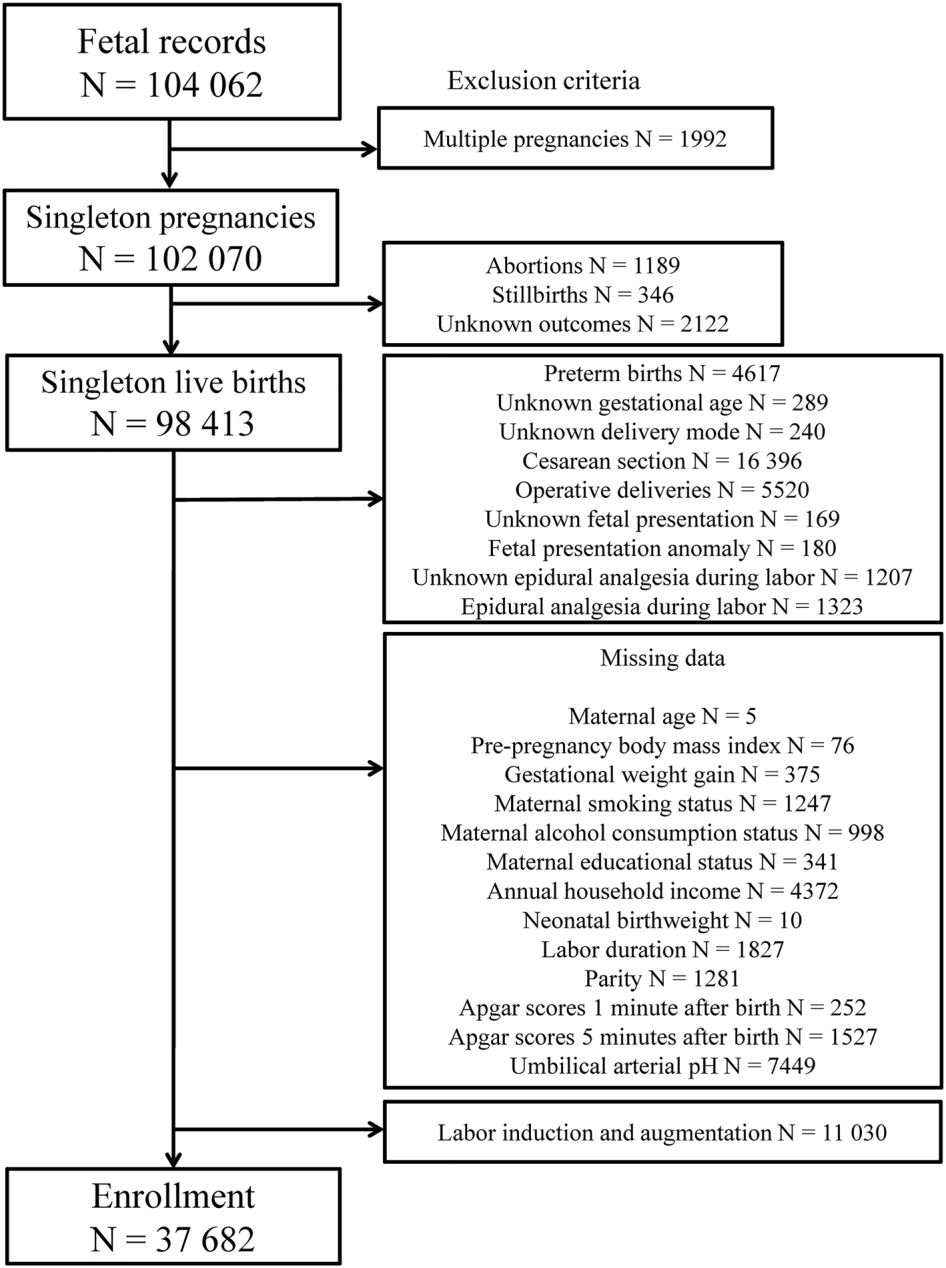
Study enrolment flowchart.

The total number of foetal records in the JECS was 104,062. Overall, 37,682 women, including 12,441 nulliparous (33.0%) and 25,241 multiparous (67.0%) women, met the inclusion criteria (Fig. 1).

### Population characteristics

Table 1 summarises the maternal characteristics and obstetric outcomes according to LD status in nulliparous and multiparous women. Pre-pregnancy BMI, GWG, and neonatal birthweight were significantly higher in women with longer LD in both nulliparous and multiparous women. The proportion of women with UmA-pH < 7.2 was significantly higher among women with longer LD in both nulliparous and multiparous women.

**Table 1:** Maternal characteristics and obstetric outcomes according to labour duration status after stratification by parity.

Parity	Nulliparous (*N* = 12,441)		Multiparous (*N* = 25,241)	
Labour duration	< 10 h	≥ 10 h	< 5 h	≥ 5 h
Variable	*N* = 6597	*N* = 5844	*N* = 13,342	*N* = 11,899
Maternal age (years), mean (SD)	29.1 (5.0)	29.1 (4.8)	32.0 (4.6)	31.7 (4.6)
Pre-pregnancy BMI (kg/m^2^), median (IQR)	20.0 (18.8–21.7)	20.2 (18.9–21.9)	20.4 (19.1–22.2)	20.6 (19.1–22.6)
GWG (kg), mean (SD)	10.5 (3.8)	10.9 (4.1)	10.0 (3.7)	10.4 (7.3)
Maternal smoking during pregnancy, % (*n*)	3.3 (216)	3.9 (225)	4.8 (642)	5.1 (608)
Maternal alcohol consumption, % (*n*)	1.3 (89)	1.1 (67)	3.9 (520)	3.7 (440)
**Maternal educational status, % (*n*)**				
< 10 years	3.8 (248)	3.4 (201)	4.8 (647)	5.2 (615)
10–12 years	29.2 (1925)	29.3 (1714)	31.3 (4176)	33.0 (3931)
13–16 years	65.6 (4329)	65.5 (3830)	62.4 (8331)	60.6 (7208)
≥ 17 years	1.4 (95)	1.7 (99)	1.4 (188)	1.2 (145)
**Annual household income, % (*n*)**				
< 2,000,000 JPY	5.9 (388)	6.0 (353)	5.2 (699)	5.6 (672)
2,000,000–5,999,999 JPY	66.8 (4406)	66.4 (3881)	68.6 (9154)	69.9 (8322)
6,000,000–9,999,999 JPY	23.4 (1543)	23.1 (1351)	22.0 (2940)	20.5 (2443)
≥ 10,000,000 JPY	3.9 (260)	4.4 (259)	4.1 (549)	3.9 (462)
Neonatal birth weight (g), mean (SD)	2965 (333)	3041 (328)	3087 (351)	3129 (353)
Hypertensive disorders of pregnancy, % (*n*)	2.1 (139)	1.7 (102)	1.0 (133)	1.0 (121)
Apgar scores < 7 at 1 min after birth, % (*n*)	1.0 (65)	1.1 (67)	0.7 (95)	0.9 (102)
Apgar scores < 7 at 5 min after birth, % (*n*)	0.3 (19)	0.4 (25)	0.2 (28)	0.2 (27)
UmA-pH < 7.2, % (*n*)	6.4 (422)	8.9 (520)	4.1 (545)	4.8 (570)
UmA-pH < 7.1, % (*n*)	1.0 (66)	1.5 (85)	0.6 (81)	0.8 (92)

BMI; body mass index, GWG; gestational weight gain, IQR, interquartile range; JPY, Japanese yen; SD, standard deviation; UmA, umbilical artery.

### Results of multiple logistic regression analyses

Table 2 summarises the cORs, aORs, and 95% CIs for low neonatal Apgar scores and foetal acidosis in women with over-median LD, based on stratification (1). No significant association was found between over-median LD and low neonatal Apgar scores at 1- and 5-min incidences. The aORs for UmA-pH < 7.2 and < 7.1 in nulliparous women with over-median LD were 1.43 (95 CI 1.25–1.63) and 1.42 (95% CI 1.02–1.96), respectively, whereas the adjusted OR for UmA-pH < 7.2 in multiparous women with over-median LDs was 1.19 (95% CI 1.05–1.34).

**Table 2:** Odds ratios for low neonatal Apgar scores and foetal acidosis in nulliparous and multiparous participants with over-median labour duration.

	Apgar scores < 7 at 1 min after birth	Apgar scores < 7 at 5 min after birth	UmA-pH < 7.2	UmA-pH < 7.1
	Odds ratios (95% CI)			
**Nulliparous**				
LD < 10 h, *N* = 6597	Ref	Ref	Ref	Ref
LD ≥ 10 h, *N* = 5844				
cORs	1.17 (0.83–1.64)	1.49 (0.82–2.70)	1.43 (1.25–1.63)	1.46 (1.06–2.02)
aORs	1.22 (0.86–1.72)	1.54 (0.84–2.81)	1.43 (1.25–1.63)	1.42 (1.02–1.96)
**Multiparous**				
LD < 5 h, *N* = 13,342	Ref	Ref	Ref	Ref
LD ≥ 5 h, *N* = 11,899				
cORs	1.21 (0.91–1.60)	1.08 (0.64–1.84)	1.18 (1.05–1.33)	1.28 (0.95–1.72)
aORs	1.22 (0.92–1.62)	1.04 (0.61–1.77)	1.19 (1.05–1.34)	1.32 (0.98–1.78)

Maternal age, pre-pregnancy body mass index, gestational weight gain, maternal smoking status, maternal alcohol consumption status, maternal educational status, annual household income, neonatal birth weight, and presence of hypertensive disorders of pregnancy were used as confounding factors.

aORs, adjusted odds ratios; CI, confidence interval; cORs, crude odds ratios; LD, labour duration; Ref, reference; UmA, umbilical artery.

Table 3 summarises the aORs and 95% CIs for foetal acidosis in women in each LD group, based on stratification (2). The aORs for UmA-pH < 7.2 increased in nulliparous women with each over-median LD category that reached a plateau in those with LD ≥ 13. Furthermore, the aORs for UmA-pH < 7.2 and UmA-pH < 7.1 were increased in multiparous women with LDs of 8–14 h and 11–14 h, respectively, albeit not in a dose–response manner.

**Table 3:** Adjusted odds ratios for foetal acidosis in nulliparous and multiparous women with each labour duration category in over-median labour duration.

	UmA-pH < 7.2	UmA-pH < 7.1
	aORs (95% CI)	
**Nulliparous**		
LD < 10.0 h, *N* = 6597	Ref	Ref
LD 10.0–12.9 h, *N* = 2064	1.26 (1.04–1.52)	1.24 (0.78–1.97)
LD 13.0–15.9 h, *N* = 1349	1.53 (1.24–1.89)	1.53 (0.93–2.51)
LD 16.0–18.9 h, *N* = 833	1.47 (1.14–1.90)	1.53 (0.84–2.79)
LD ≥ 19.0 h, *N* = 1598	1.54 (1.27–1.87)	1.49 (0.93–2.38)
**Multiparous**		
LD < 5.0 h, *N* = 13,342	Ref	Ref
LD 5.0–7.9 h, *N* = 6900	1.15 (0.99–1.32)	1.17 (0.82–1.68)
LD 8.0–10.9 h, *N* = 2788	1.27 (1.05–1.53)	1.23 (0.75–2.02)
LD 11.0–13.9 h, *N* = 1239	1.43 (1.10–1.85)	2.42 (1.43–4.11)
LD ≥ 14.0 h, *N* = 972	0.99 (0.71–1.38)	1.27 (0.58–2.76)

Maternal age, pre-pregnancy body mass index, gestational weight gain, maternal smoking status, maternal alcohol consumption status, maternal educational status, annual household income, neonatal birth weight, and presence of hypertensive disorders of pregnancy were used as confounding factors.

aORs, adjusted odds ratios; CI, confidence interval; LD, labour duration; Ref, reference; UmA, umbilical artery.

In under-median LD, there was a partially significant association between the divided categories, decreased incidence of low neonatal Apgar scores at 5 min and foetal acidosis in nulliparous women, and only UmA-pH < 7.2 in multiparous women without dose–response association (data not shown).

### Results of receiver operating characteristic curve analysis

The cut-off LD values for the prediction of foetal acidosis (UmA-pH < 7.2) were 598 min (sensitivity, 55.8%; specificity, 53.2%; and area under the curve [AUC], 0.554) in nulliparous women and 357 min (sensitivity, 41.4%; specificity, 63.9%; and AUC, 0.526) in multiparous women.

## Discussion

### Main findings

This study revealed an association between over-median LD in nulliparous and multiparous women and increased foetal acidosis incidence, without any statistically significant association between over-median LD and low neonatal Apgar scores incidence. The association between over-median LD and increased incidence of foetal acidosis manifested in a plateau in nulliparous women with LD ≥ 13 h and without dose-dependent association in multiparous women. The LD cut-off values established in this study had low sensitivity and specificity. A strength of the present study is evaluating the association between LD and the incidence of low neonatal Apgar scores and foetal acidosis in women with low-risk spontaneous deliveries in a nationwide population.

### Interpretations

This study, which included a large number of women with spontaneous deliveries without labour induction and augmentation with several novel confounding factors, makes a significant contribution to the literature because it presents strong findings confirming a significant association between over-median LD and the foetal acidosis incidence. These findings are consistent with those of previous studies showing that prolonged LD was associated with an increased incidence of neonatal morbidity^9,10^. Nonetheless, several studies have shown that even women with prolonged LD can achieve a successful vaginal delivery^21–23^ and have indicated that prolonged first and second stages of labour are not associated with an increased incidence of low neonatal Apgar scores and foetal acidosis^11–15^. One possible reason for this discrepancy is the difference in the definition of prolonged LD. This study defined over-median LD based on median values, so a larger number of women were assigned to a longer LD in this study than in previous studies. Furthermore, the present study analysed a much larger study population and removed the influence of multiple delivery settings by including only full-term and spontaneous deliveries with cephalic presentation and without epidural analgesia or labour induction and augmentation. This enabled us to elucidate the association between LD and the incidence of low neonatal Apgar scores and foetal acidosis in a limited population of women with spontaneous deliveries. Conversely, in this setting, only a few women had infants with low Apgar scores. This may be the reason for the lack of a significant association between LD and the low neonatal Apgar scores incidence. Therefore, further studies should clarify the association of LD with Apgar scores ≤ 3 and UmA-pH < 7.0 in a population with more severe conditions.

Uterine contractions in labour cause a 60% reduction in uteroplacental perfusion, leading to transient foetal and placental hypoxia with subsequent foetal asphyxia and acidosis^24^. Although most foetuses can tolerate this reduction in placental perfusion, some cannot^24^. Furthermore, although uterine contractions result in foetal O_2_ reduction of approximately 25%^24^, most healthy foetuses can withstand this and even cope with an O_2_ reduction of up to 50%^24^. However, extended LD may cause recurrent, prolonged, and excessive reduction of uteroplacental perfusion, which may exceed the ability of the foetus to tolerate the hypoxia and lead to foetal asphyxia and acidosis. Furthermore, because labour is affected by inflammatory status in the myometrium, cervix, and foetal membranes^25–27^, prolonged LD may be associated with an inflammatory condition that may also affect foetal asphyxia and acidosis, along with the effects of labour dystocia on histological chorioamnionitis and funisitis^28^.

Although Japanese women have a leaner physique than western women, Suzuki et al. created a labour curve using data from 2,369 Japanese nulliparous women and obtained results similar to those of Zhang et al., who studied a multi-ethnic group of 1,162 nulliparous women^29,30^. However, given that Tuck et al. and Greenberg et al. reported that Asian women had a longer second stage of labour than Caucasian women^31,32^, an Asia-centred analysis regarding the association between LD and the incidence of low neonatal Apgar scores and foetal acidosis was necessary. Thus far, no nationwide study has attempted to determine a safe LD for Asian women so that the incidence of low neonatal Apgar scores and foetal acidosis can be reduced.

However, a cut-off LD value of approximately 10 h in nulliparous women had low sensitivity, specificity, and AUC values, making it a less useful tool to universally determine foetal risks, because detailed clinical scenarios were not considered in this study. In contrast, it can pose a potential risk of foetal acidosis because aORs were also significantly increased in nulliparous women with LD ≥ 10 h and reached a plateau with LD ≥ 13 h. Conversely, in multiparous women, the cut-off LD value could not predict the incidence of foetal acidosis, and aORs did not significantly increase in a dose–response manner, probably because multiparous women tend to have a lower risk of a complicated birth than nulliparous women^33^. Similarly, we speculate that the reason for the lack of significant association between over-median LD (≥ 5 h) and UmA-pH < 7.1 in multiparous participants is the much lower occurrence of UmA-pH < 7.1 in multiparous women than in nulliparous women. Although over-median LD is a clinical sign for caution of foetal acidosis, establishment of optimal LD to reduce the incidence of low neonatal Apgar scores and foetal acidosis might be contentious, because of the considerable variation from foetus to foetus in the ability to tolerate a specific length of labour. Further studies should be performed to clarify the association between LD and foetal and neonatal outcomes with detailed stratified analysis based on delivery settings.

### Limitations

This study has some limitations. First, women might have experienced their labour onset in various ways, which made precise evaluation of LD difficult^34,35^, and the JECS data set could not discriminate the stage of labour (first or second stage). Discrimination between the stages of labour would lead to different results regarding the association. Careful interpretation of the instability of LD is needed. Second, there was no information regarding the umbilical cord acid–base data; therefore, respiratory acidosis could not be discriminated from metabolic acidosis. All cord arterial pH samples were generally immediately collected in all institutions, although there was no unified protocol to collect them. Additionally, no information was available on cord vein analysis in this data set. Moreover, no quality assessment was made regarding the validity of the results. Therefore, careful interpretation of the results is warranted. Third, the study did not account for the detailed clinical scenario, such as amniotic fluid levels, foetal malrotation, cervical dilatation, detailed data regarding foetal heart rate monitoring, genital bleeding during labour, time point of rupture of membranes, intrauterine infection, presence of support for dystocia including uterine fundal pressure and management of shoulder dystocia, and foetal anomalies. Furthermore, no information was available regarding foetal resuscitation during labour and neonatal resuscitation after birth, making it impossible to analyse the effects of foetal resuscitation and neonatal resuscitation on neonatal outcomes. However, our multivariate analysis considered several confounding factors and included only women who had spontaneous deliveries and did not require abrupt delivery due to foetal distress, which further strengthens our findings. Finally, because complete case analysis in this study might have led to potential biases, our results should be interpreted with caution.

## Conclusions

Over-median LD was associated with an increased incidence of foetal acidosis in women with low-risk spontaneous deliveries. This association manifested as a plateau among nulliparous women with LD ≥ 13 h. This association was not dose-dependent in multiparous women. Meanwhile, over-median LD was not significantly associated with the incidence of low neonatal Apgar scores. More than 10 h of LD posed potential risks of foetal acidosis in nulliparous women in the JECS; however, the cut-off values of LD for predicting the incidence of foetal acidosis exhibited low sensitivity and specificity. Further studies should be performed to clarify the association between LD and foetal and neonatal outcomes with detailed stratified analysis based on delivery settings.

## Data Availability

Data are unsuitable for public deposition due to ethical restrictions and legal framework of Japan. It is prohibited by the Act on the Protection of Personal Information (Act No. 57 of 30 May 2003, amendment on 9 September 2015) to publicly deposit the data containing personal information. Ethical Guidelines for Epidemiological Research enforced by the Japan Ministry of Education, Culture, Sports, Science and Technology and the Ministry of Health, Labour and Welfare also restrict the open sharing of the epidemiologic data. All inquiries about access to data should be sent to: jecs-en@nies.go.jp. The person responsible for handling enquiries sent to this e-mail address is Dr. Shoji F. Nakayama, JECS Programme Office, National Institute for Environmental Studies.
